# Supplementary material for: Heat adaptation of phage T7 under an extended genetic code
Source: Virus Evol. 2021 Dec 1;7(2):veab100. doi: 10.1093/ve/veab100 (PMC8923235; doi:10.1093/ve/veab100)
Supplement: veab100_Supp [file veab100_supp.zip › Sequencing_Analysis.zip!/Sequencing_Analysis/A1/evidence/JC_SIDE_2_51.html]

BRESEQ :: Evidence


| New junction evidence | | | | | | | | | | | |
| --- | --- | --- | --- | --- | --- | --- | --- | --- | --- | --- | --- |
|  | | seq id | position | reads (cov) | reads (cov) | score | skew | freq | annotation | gene | product |
| \* | ? | NC\_001604.1 | 295 = | 3899 (1.260) | 25 (0.010) | 15/560 | 36.7 | 0.68% | intergenic (–/–) | *–/–* | –/– |
|  | ? | NC\_001604.1 | = 281 | 3655 (1.270) |  |  |  |  | intergenic (–/–) | *–/–* | –/– |
| Rejected: Coverage evenness skew score above cutoff. | | | | | | | | | | | |
| Rejected: Frequency below/above cutoff threshold. | | | | | | | | | | | |

Only 100 of 3838 total aligned reads displayed.  

`‑‑‑‑‑‑‑‑TCTCACAGTGTACGGACCTAAAGTTCCCCCATAGGGGGTACCTAAAGCCCAGCCAATCACCTAAAGTCAACCTTCGGTTGACCTTGAGGGTTCCCTAAGGGTTGGGGATGACCCTTGGGTTTGTCTTTGGGTGTTACCTTGAGTGTCTCTCTGTGTCCCTATCTGTTACAGTCTCCTAAAGTATCCTCCTAAAGTCACCTCCTAACGTCCATCCTAAAGCCAACACCTAAAGCCTACACCTAAAGACCCATCAAGTCAACGCCTATCTTAAAGTTTAAACATAAAGACCAGACCTAAAGACCAGACCTAAAGACAC‑TACATAAAGACCAGACCTAAAGACGCCTTGTTGTTAGCCATAAAGTGATAACCTTTAATCATTGTCTTTATTAATACAACTCACTATAAGGAGAGACAACTTAAAGAGACTTAAAAGATTAATTTAAAATTTATCAAAAAGAGTATTGACTTAAAGTCTAACCTATAGGATACTTACAGCCATCGAGAGGGACACGGCGAATAGCCATCCCAATCGACACCGGGGTCAACCGGATAAGTAGACAGCCTGATAAGTCGCACGAAAA  >  NC_001604.1/1‑581`  

                                                                                                                                                                                                                                                                                                `|`                                                                                                                                                                                                                                                                                                               
`gtgtataagagacaggtgtACGGACCTAAAGTTCCCCCATA‑GGGGTACCTAAAGCCCAGCCAATCACCTAAAGTCAACCTTCGGTTGACCTTGAGGGTTCCCTAAGGGTTGGGGATGACCCTTGGGTTTGTCTTTGGGTGTTACCTTGAGTGTCTCACTGTGTCCCTATCTGTTACAGTCTCCTAAAGTATCCTCCTAAAGTCACCTCCTAACGTCCATCCTAAAGCCAACACCTAAAGCCTACACCTAAAGACCCATCAAGTCAACGCCTATCTTAAAGTTTAAACAtaaagaccagacc                                                                                                                                                                                                                                                                                                  <  1:19968/286‑1 (MQ=255)`  
     `tgtataagagacagaCGGACCTAAAGTTCCCCCATA‑GGGGTACCTAAAGCCCAGCCAATCACCTAAAGTCAACCTTCGGTTGACCTTGAGGGTTCCCTAAGGGTTGGGGATGACCCTAGGGTTTGTCTTTTGGTGTTACCTTGAGTGTCTCTCTGTGTCCCTATCTGTTACAGTCTCCTAAAGTATCCTCCTAAAGTCACCTCCTAACGTCCATCCTAAAGCCAACACCTAAAGCCTACACCTAAAGACCCATCAAGTCAACGCCTATCTTAAAGTTTAAACAtaaagaccagacctaaag                                                                                                                                                                                                                                                                                             <  1:451271/287‑1 (MQ=255)`  
     `atgggtataagagacaggACCTAAAGTTCCCCCATA‑GGGGTACCTAAAGCCCAGCCAATCACCTAAAGTCAACCTTCGGTTGACCTTGAGGGTTCCCTAAGGGTTGGGGATGACCCTTGGGTTTGTCTTTGGGTGTTACCTTGAGTGTCTCTCTGTGTCCCTATCTGTTACAGTCTCCTAAAGTATCCTCCTAAAGTCACCTCCTAACGTCCATCCTAAAGCCAACACCTAAAGCCTACACCTAAAGACCCATCAAGTCAACGCCTATCTTAAAGTTTAAACAtaaagaccagacctaaag                                                                                                                                                                                                                                                                                             <  1:390720/285‑1 (MQ=255)`  
        `gggagatgtgtataagagacaggTTCCCCCATA‑GGGGTACCTAAAGCCCAGCCAATCACCTAAAGTCAACCTTCGGTTGACCTTGAGGGTTCCCTAAGGGTTGGGGATGACCCTTGGGTTTGTCTTTGGGTGTTACCTTGAGTGTCTCTCTGTGTCCCTATCTGTTACAGTCTCCTAAAGTATCCTCCTAAAGTCACCTCCTAACGTCCATCCTAAAGCCAACACCTAAAGCCTACACCTAAAGACCCATCAAGTCAACGCCTATCTTAAAGTTTAAACAtaaagaccagacctaaagacc                                                                                                                                                                                                                                                                                          <  1:277833/279‑1 (MQ=255)`  
        `gagacaggtgtACTGACCTAAAGTTCCCCCATA‑GGGGTACCTAAAGCCCAGCCAATCACCTAAAGTCAACCTTCGGTTGACCTTGAGGGTTCCCTAAGGGTTGGGGATGACCCTTGGGTTTGTCTTTGGGTGTTACCTTGAGTGTCTCTCTGTGTCCCTATCTGTTACAGTCTCCTAAAGTATCCTCCTAAAGTCACCTCCTAACGTCCATCCTAAAGCCAACACCTAAAGCCTACACCTAAAGACCCATCAAGTCAACGCCTATCTTAAAGTTTAAACAtaaagaccagacctaaagacc                                                                                                                                                                                                                                                                                          <  1:29435/294‑1 (MQ=255)`  
        `aagagacaggtccGGACCTAAAGTTCCCCCATA‑GGGGTACCTAAAGCCCAGCCAATCACCTAAAGTCAACCTTCGGTTGAACTTGAGGGTTCCCTAAGGGTTGGGGATGACCCTTGGGTTTGTCTTTGGGTGTTACCTTGAGTGTCTCTCTGTGTCCCTATCTGTTACAGTCTCCTAAAGTATCCTCCTAAAGTCACCTCCTAACGTCCATCCTAAAGCCAACACCTAAAGCCTACACCTAAAGACCCATCAAGTCAACGCCTATCTTAAAGTTTAAACAtaaagaccagacctaaagacc                                                                                                                                                                                                                                                                                          <  1:256492/289‑1 (MQ=255)`  
               `gtgtACGGACCTAAAGTTCCCCCATA‑GGGGTACCTAAAGCCCAGCCAATCACCTAAAGTCAACCTTCGGTTGACCTTGAGGGTTCCCTAAGGGTTGGGGATGACCCTTGGGTTTGTCTTTGGGTGTTACCTTTAGTGTCTCTCTTTTTCCCTATCTGTTACAGTCTCCT‑AAGTATCATCCTAAATTCACCTCCTAACGTCCATCCTAAATCCAACACCTAAAGCCTACCCCTAAAGACCCATCAAGTAAACGCCTATCTTAAAGTTTCAACATACagaccagacctaaagaccagaactaa                                                                                                                                                                                                                                                                                  >  1:195756/1‑301 (MQ=25)`  
               `gtgtACGGACCTAAAGTTCCCCCATA‑GGGGTACCTAAAGCCCAGCCAATCACCTAAAGTCAACCTTCGGTTGACCTTGAGGGTTCCCTAAGGGTTGGGGATGACCCTTGGGTTTGTCTTTGGGTGTTACCTTGAGTGTCTCTCTGTGTCCCTATCTGTTACAGTCTCCTACAGTATCCTCCTAAAGTCACCTCCTACCGTCCATCCTAAAGCCAACACCTAAAGCCTACACCTAAAGACCCATCAAGTCAACGCCTATCTTAAAGTTTAAACATAaagcccagccctaaagaccagaccta                                                                                                                                                                                                                                                                                   >  1:434658/1‑301 (MQ=255)`  
               `gtgtACGGACCTAAAGTTCCCCCATA‑GGGGTACCTAAAGCCCAGCCAATCACCTAAAGTCAACCTTCGGTTGACCTTGAGGGTTCCCTAAGGGTTGGGGATGACCCTTGGGTTTGTCTTTGGGTGTTACCTTGAGTGTCTCTCTGTGTCCCTATCTGTTACAGTCTCCTACAGTATCCTCCTAAAGTCACCTCCTAACGTCCATCCTACAGCCAACACCTAAAGCCTACACCTAAAGACCCATCAAGTCAACGCCTATCTTAAAGTTTAAACATAaagaccagacctaaagaccagaccta                                                                                                                                                                                                                                                                                   >  1:71109/1‑301 (MQ=255)`  
               `gtgtACGGACCTAAAGTTCCCCCATA‑GGGGTACCTAAAGCCCAGCCAATCACCTAAAGTCAACCTTCGGTTGACCTTGAGGGTTCCCTAAGGGTTGGGGATGACCCTTGGGTTTGTCTTTGGGTGTTACCTTGAGTGTCTCTCTGTGTCCCTATCTGTTACAGTCTCCTAAAGTATCCTCCTAAAGTCACCTCCTAACGTCCATCCTAAAGCCAACACCTAAAGCCTACACCTAAAGACCCATCAAGTCAACGCCTATCTTAAAGTTTAAACATAaagaccagacctaaagaccaaaccta                                                                                                                                                                                                                                                                                   >  1:203600/1‑301 (MQ=255)`  
               `gtgtACGGACCTAAAGTTCCCCCATA‑GGGGTACCTAAAGCCCAGCCAATCACCTAAAGTCAACCTTCGGTTGACCTTGAGGGTTCCCTAAGGGTTGGGGATGACCCTTGGGTTTGTCTTTGGGTGTTACCTTGAGTGTCTCTCTGTGTCCCTATCTGTTACAGTCTCCTAAAGTATCCTCCTAAAGTCACCTCCTAACGTCAATCCTAAAGCCAACACCTAAAGCCTACACCTAACGACCCATCAAGTCAACGCCTATCTTAAAGTTTAAACAtaaagaccagacctaaagaccagaccct                                                                                                                                                                                                                                                                                   >  1:429854/1‑299 (MQ=255)`  
               `gtgtACGGACCTAAAGTTCCCCCATA‑GGGGTACCTAAAGCCCAGCCAATCACCTAAAGTCAACCTTCGGTTGACCTTGAGGGTTCCCTAAGGGTTGGGGATGAACCTTGGGTTTGTCTTTGGGTGTTACCTTGAGTGTCTCTCTGTGTCCCTATCTGTTACAGTCTCCTAAAGTATCCTCCTAAAGTCACCTCCTAACGTCCATCCTAAAGCCAACACCTAAAGCCTACACCTAAAGACCCATCAAGTCAACGCCTATCTTAAAGTTTAAACATAaagacaagacctaaagaccagaccta                                                                                                                                                                                                                                                                                   >  1:119079/1‑301 (MQ=255)`  
                 `gtACGGACCTAAAGTTCTCCCATA‑GGGGTACCTAAAGCCCAGCCAATCACCTAAAGTCAACCTTCGGTTGACCTTGAGGGTTCCCTAAGGGTTGGGGATGACCCTTGGGTTTGTCTTTGGGTGTTACCTTGAGTGTCTTTCTGTGTCCCTATCTGTTACAGTCTCCTAAAGTCTCCTCCTAAAGTCACCTCCTAACGTCAATCCTAAAGCCAACACCTAACTCCTCCACCTAAAGACCCATCAAGTCCACGCCTATCTTAAAGTTTCAACATAAAtaccagacctaaagacccaacctaaa                                                                                                                                                                                                                                                                                 >  1:386794/1‑301 (MQ=25)`  
                 `gtACGGACCTAAAGTTCCCCCATA‑GGGGTACCTAAAGCCCAGCCAATCACCTAAAGTCAACCTTCGGTTGACATTGAGGGTTCCCTAAGGGTTTGGGATGACCCTTGGGTTTGTCTTTGGGTGTTACCTTGAGTGTCTCTCTGTGTCCCTATCTGTTACAGTATCCTAAAGTATCCTACTAAAGGCACCTCCTAACGTCCATCCTAAAGACAACACCAAAAGCATACACCTAAAGACCCATAAAGTCAACGACTATCTTAAAGTTTAAACAAAAAgaacatacctaaagaccagacctaaa                                                                                                                                                                                                                                                                                 >  1:381961/1‑301 (MQ=25)`  
                    `cGGGCCTAAAGTTCCCCCATA‑GGGGTACCTAAAGCCCAGCCAATCCCCTAAAGTCAACCTTCGGTTGACCTTGAGGGTTCCCTAAGGGTTGGGGATGACCCTTGGGTTTGTCTTTGGGTGTTACCTTGAGTGTCTCTCTGTGTCCCTATCTGTTACAGTCTCCTAAAGTATCCTCCTAAAGTCACCTCCTAACGTCCATCCTAAAGCCAACACCTAAAGCCTACACCTAAAGACCCATCAAGTCAACGCCTATCTTAAAGTTTAAACATAAAGACcagacctaaagaccagacctaaagac                                                                                                                                                                                                                                                                              <  1:367267/301‑1 (MQ=255)`  
                             `aGTTCCCCCATA‑GGGGTACCTAAATCCCAGCCAATCACCTAAAGTCAACCTTCGGTTGACCTTGAGGGTTCCCTAAGGGTTGGGGATGACCCTTGGGTTTGTCTTTGGGTGTTACCTTTAGTGTCTCTCTGTGTCCCTATCTGTTACAGTCTCCTAAAGTATCTTCCTAAAGTCACCTCCTAACGTCCATCCTAAAGCCAACACCTAAAGCCTACACCTAAAGACCCATCAAGTCAACGCCTATCTTAAAGTTTAAACATAAAGACCAGACCTAAAGACCAGACCTAAAGACAC‑TACATaa                                                                                                                                                                                                                                                                    <  1:232366/301‑1 (MQ=255)`  
                                 `gtgtataagagacagccTAAAGCCCAGCCAATCACCTAAAGTCAACCTTCGGTTGACCTTGAGGGTTCCCTAAGGGATGGGGATTGCCCTTGGGTTTGTCTTTGGGTGTTACCTTGAGTGTCTCTCTGTGTCCCTATCTGTTACAGTCTCCTAAAGTATCCTCCTAAAGTCACCTCCTAACGTCCATCCTAAAGCCAACACCTAAAGCCTACACCTAAAGACCCATCAAGTCAACGCCTATCTTAAAGTTTAAACATAAAGACCAGACCTAAAGACCAGACCTAAAGACAC‑TACATAAaga                                                                                                                                                                                                                                                                 <  1:26603/286‑1 (MQ=255)`  
                                              `tACCTAAATCCCAGCCAATCACCTAAAGTCAACCTTCGGTTGACCTTGAGGGTTCCCTAAGGGTTGCGGATGACCCTTGGGTTTGTCTTTGGGTGTTACCTTGAGTGTCTCTCTGTGTCCCTATCTGTTACAGTCTCCTAAAGTATCCTCCTAAAGTCACCTCCTAACGTCCATCCTAAAGCCAACACCTAAAGCCTACACCTAAAGACCCATCAAGTCAACGCCTATCTTAAAGTTTAAACATAAAGACCAGACCTAAAGACCAGACCTAAAGACAC‑TACATAAAGACCAGACCTAAAGa                                                                                                                                                                                                                                                    <  1:218442/301‑1 (MQ=255)`  
                                                `ccTAAAGCCCAGCCAATCACCTAAAGTCAACCTTCGGTTGACCTTGAGGGTTCCCTAAGGGTTGGGGATGACCCTTGGGTTTGTCTTTGGGTGTTACCTTGAGTGTCTCTCTGTGTCCCTATCTGTTACAGTCTCCTAAAGTATCCTCCTAAAGTCACCTCCTAACGTCCATCCTAAAGCCAACACCTAAATCCTACACCTAAAGACCCATCAAGTCAACGCCTATCTTAAAGTTTAAACATAAAGACCAGACCTAAAGACCAGACCTAAAGACAC‑TACATAAAGACCAGACCTAAAGACg                                                                                                                                                                                                                                                  >  1:472763/1‑301 (MQ=255)`  
                                                `ccTAAAGCCCAGCCAATCACCTAAAGTCAACCTTCGGTTGACCTTGAGGGTTCCCTAAGGGTTGGGGATGACCCTTGGGTTTGTCTTTGGGTGTTACCTTGAGTGTCTCTCTGTGTCCCTATCTGTTACAGTCTCCTAAAGTATCCTCCTAAAGTCACCTCCTAACGTCCATCCTAAAGCCAACACCTAAAGCCTACACCTAAAGACCCATCAAGTCAACGCCTATCTTAAAGTTTAAACATAAAGACCAGACCTAAAGACCAGACCTAAAGACAC‑TACATAAaaacaagaccctgtctct                                                                                                                                                                                                                                                  >  1:328404/1‑293 (MQ=255)`  
                                                        `ttgtataagatacggaaGTCAACCTTCGGTTTACCTTTAGGGTTCCCTAAGGGTTGTTGATGACCCTTGTGTTTGTCTTTGGGTGTTACCTTGAGTGTCTCTCTGTGTCCCTATCTGTTACAGTCTCCTAAAGTATCCTCCTAAAGTCACCTCCTAACGTCCATCCTAAAGCCAACACCTAAAGCCTACACCTAAAGACCCATCAAGTCAACGCCTATCTTAAATTTTAAACATAAAGACCAGACCTAAAGACCAGACCTAAAGACAC‑TACATAAAGACCAGACCTAAAGACGCCttgttg                                                                                                                                                                                                                                          <  1:312505/286‑1 (MQ=38)`  
                                                        `ccAGCCAATCCCCTAAAGTCAACCTTCGGTTGACCTTGAGGGTTCCCTAAGGGTTGGGGATGACCCTTGGGTTTGTCTTTGGGTGTTACCTTGAGTGTCTCTCTGTGTCCCTATCTGTTACAGTCTCCTAAAGTATCCTCCTAAAGTCACCTCCTAACGTCCATCCTAAAGCCAACACCTAAAGCCTACACCTAAAGACCCATCAAGTCAACGCCTATCTTAAAGTTTAAACATAAAGACCAGACCTAAAGACCAGACCTAAAGACAC‑TACATAAAGACCAGACCTAAAGACGCCttgttg                                                                                                                                                                                                                                          <  1:152540/301‑1 (MQ=255)`  
                                                             `tataagagacaggTCAACCTTCGGTTGACCTTGAGGGTTCCCTAAGGGTTGGGGATGACCCTTGGGTTTGTCTTTGGGTGTTACCTTGAGTGTCTCTCTGTGTCCCTATCTGTTACAGTCTCCTAAAGTATCCTCCTAAAGTCACCTCCTAACGTCCATCCTAAAGCCAACACCTAAAGCCTACACCTAAAGACCCATCAAGTCAACGCCTATCTTAAAGTTTAAACATAAAGACCAGACCTAAAGACCAGACCTAAAGACAC‑TACATAAAGACCAGACCTAAAGACGCCTTGTTGTTAGc                                                                                                                                                                                                                                     <  1:480850/289‑1 (MQ=255)`  
                                                               `aTCACCTAAAGTCAACCTTCGGTTGACCTTGAGGGTTCCCTAAGGGTTGGGGATGACCCTTAGGTTTGTCTTTGGGTGTTACCTTGAGTGTCTCTCTGTGTCCCTATCTGTTACAGTCTCCTAAAGTATCCTCCTAAATTCACCTCCTAACGTCCTTCCTAAACCCAACCCCTAAAGCTTACACCTAAAAACCCATAAATACAACGCCTTCTTTAAATTTCAAACATAAAAACAAAACCTAAAGACCAAACCTAATG‑CACTTACAAAAACACCAACCCTAAAGATCCCttgtctgtgccct                                                                                                                                                                                                                                   >  1:358257/1‑292 (MQ=21)`  
                                                                 `cACCTAAAGTCAACCTTCGGTTGACCTTGAGGGTTCCCTAAGGGTTGGGGATGACCCTTGGGTTTGTCTTTGGGTGTTACCTTGAGTGTCTCTCTGTGTCCCTATCTGTTACAGTCTCCTAAAGTATCCTCCTAAAGTCACCTCCTAACGTCCATCCTAAAGCCAACACCTAAAGCCTACACCTAAAGACCCATCAAGTCAACGCCTATCTTAAAGTTTAAACATAAAGACCAGACCTAAAGACCAGACCTAAAGACAC‑TACATAAAGACCAGACCTAAAGACGCCttgttgctgactctt                                                                                                                                                                                                                                 >  1:261320/1‑292 (MQ=255)`  
                                                                            `aaCCTTCGGTTGACCTTGAGGGTTCCCTAAGGGTTGGGGATGACCCTTGGGTTTGTCTTTGGGTGTTACCTTGAGTGTCTCTCTGTGTCCCTATCTGTTACAGTCTCCTAAAGTATCCTCCTAAAGTCACCTCCTAACGTCCATCCTAAAGCCAACATCTAAAGCCTACACCTAAAGACCCATCAAGTCAACGCCTATCTTAAAGTTTAAACATAAAGACCACACCTAAAGACCAGACCTAAAGACAC‑TACATAAAGACCAGACCTAAAGACGCCTTGTTGTTAGCCATAAAGTGATAAcc                                                                                                                                                                                                                      <  1:138922/301‑1 (MQ=255)`  
                                                                                  `cGGTTGACCTTGAGGGTTCCCTAAGGGTTGGGGATGACCCTTGGGTTTGTCTTTGGGTGTTACCTTGAGTGTCTATCTGTGTCCCTATCTGTTACAGTCTCCTAAAGTATCCTCCTAAAGTCACCTCCTAACGTCCATCCTAAAGCCAACACCTAAAGCCTACACCTAAAGACCCATCAAGTCAACGCCTATCTTAAAGTTTAAACATAAAGACCAGACCTAAAGACCAGACCTAAAGACAC‑TACATAAAGACCAGACCTAAAGACGCCTTGTTGTTAGCCATAAAGTGATAACCTTTAAt                                                                                                                                                                                                                <  1:269436/301‑1 (MQ=255)`  
                                                                                   `ggatGACCTGGAGGGTAAGCAAAGGGTTGGGGATGAAGGTTGGGATTGTCGATGGGTGTGACCATGAATGAAACACAGTGTACATAAATGTTACAGTCGAATAAAGTATCCACATAAAGGCAGCTCCTAAAGTCCATCCAAAAGAAAACACCTAAAAGCAACACCTAAAGACAAATCAAGTAAAAGAGTATCTTAAAGAAGAAACATAAAGACAAGACCTAAAGAAAAGACCAAAAGACAC‑TACATAAAGACCAGACCTAAAGACGCCTTGTTGTTAGCCATAAAGTGATAACCTTTAATc                                                                                                                                                                                                               <  1:209666/298‑1 (MQ=255)`  
                                                                                   `atgtgtataagagacagcccTAAGGGTTGGGGATGACCCTTGGGTTTGTCATTGGGTGTTACCTTGAGTGTCTCTCTGTGTCCCTATCTGTTACAGTCTCCTAAAGTATCCTCCTAAAGTCACCTCCTAACGTCCATCCTAAAGCCAACACCTAAAGCCTACATCTAAAGACCCATCAAGTCAACGCCTATCTTAAAGTTTAAACATAAAGACCAGACCTAAAGACCAGACCTAAAGACAC‑TACATAAAGACCAGACCTAAAGACGCCTTGTTGTTAGCCATAAAGTGATAACCTTTAATc                                                                                                                                                                                                               <  1:240892/284‑1 (MQ=255)`  
                                                                                   `agacagccTTGAGGGTTCCCTAAGGGTTGGGGATGACCCTTGGGTTTGTCTTTGGGTGTTACCTTGAGTGTCTCTCTGTGTCCCTATCAGTTACAGTCTCCTAAAGTATCCTCCGAAAGTCACCTCCTAACGTCCATCCTAAAGCCAACACCTAAAGCCTACACCTAAAGACCCATCAAGGCAACGCCTATCTTAAAGTTTAAACATAAAGACCAGACCTAAAGACCAGACCTAAAGACAC‑TACATAAAGACCAGACCTAAAGACGCCTTGTTGTTAGCCATAAAGTGATAACCTTTAATc                                                                                                                                                                                                               <  1:90579/295‑1 (MQ=255)`  
                                                                                     `ttGACCTTGGGGGGTCCCTAAGGGTTGGGGATGTCCCTTGGTTTTGTCTTTGGGTGTTACCCTGAGTGTCTCTCTGTGTCCCTATCTGTTACAGTCTCCTAAAGTATCCTCCTAAAGTCACCTCCTAACGTCCATCCTAAAGCCAACACCTAAAGCCTACACCTAAAGACCCATCAAGTCAACGCCTATCTTAAAGTTTAAACATAAAGACCAGACCTAAAGACCAGACCTAAAGACAC‑TACATAAAGACCAGACCTAAAGACGCCTTGTTGTTAGCCATAAAGTGATAACCTTTAATCAt                                                                                                                                                                                                             <  1:299225/301‑1 (MQ=255)`  
                                                                                       `gACCTTGAGTGTTCCCTAAGGGTTGGGGATGACCCTTGGGTTTGTCTTTGGGTGTTACCTTGAGTGTCTCTCTGTGTCCCTATCTGTTACAGTCTCCCAAAGTATCCTCCTAAAGTCACCTCCTAACGTCCATCCTAAAGCCAACACCTAAAGCCTACACCTAAAGACCCATCAAGTCAACGCCTATCTTAAAGTTTAAACATAAAGACCAGACCTAAAGACCAGACCTAAAGACAC‑TACATAAAGACCAGACCTAAAGACGCCTTGTTGTTAGCCATAAAGTGATAACCTTTAATCATTg                                                                                                                                                                                                           <  1:214492/301‑1 (MQ=255)`  
                                                                                       `gACCTTGAGGGTTCCCTAAGGGTTGGGGATGACCCTTGGGTTTGTCTTTGGGTGTTACCTTGAGTGTCTCTCTGTGTCCCTATCTGTTACAGTCTCCTAAAGTATCCTCCTAAAGTCACCTCCTAACGTCCATCCTAAAGCCAACACCTAAAGCCTACACCTAAAGACCCATCAAGTCAACGCCTATCTTAAAGTTTAAACATAAAGACCAGACCTAAAGACCAAACCTAAAGACAC‑TACATAAAGACCAGACCTAAAGACGCCATGTTGTTAGCCATAAAGTGAAAACCTTTAATcctgt                                                                                                                                                                                                           >  1:337159/1‑297 (MQ=255)`  
                                                                                              `gggggTCACTAAGAGGTGGGGATGACCCTTGGGGTAGTCATTGGGGGTTACATTGAATGTATCTCTGTGTCCATATCTGTTACAGTCACATAAAGTAAACACCTAAAGTAACCTCCTAACGTCCAAACTAAAGCCAACACCTAAAGCCTAAAACTAAAGAACCATCAAGTAAACGACTATCTTAAAGTTTAAACATAAAGACCAGACGTAAAGACCAGACCGAAAGACAA‑TACATAAAGACCAGAACTAAAGACGCCTTGTTGTTAGCCATAAAGTGATAACCTTTAATCATTATCTttat                                                                                                                                                                                                    <  1:485339/300‑1 (MQ=255)`  
                                                                                               `gggTTCCCTAAGGGTTGGGGATGACCCTTGGGTTTGTCTTTGGGTGTTACCTTGAGTGTCTCTCTGTGTCCCTATCTGTTACAGTCTCCTAAAGTATCCTCCTAAAGTCACCTCCTAACGTCCATCCTAAAGCCAACACCTAAAGCCTACACCTAAAGACCCATCAAGTCAACGCCTATCTTAAAGTTTAAACATAAAGACCAGACCTAAAGACCAGACCTAAAGACAC‑TACATAAAGACCAGACCTAAAGACGCCTTGTTGTTAGCCATAAAGTGATAACCTTTAATCATTGTCTttatt                                                                                                                                                                                                   <  1:439751/301‑1 (MQ=255)`  
                                                                                                   `tCCCTAAGGGTTGGGGATGACCCTTGGGTTTGTCTTTGGGTGTTACCTTGAGTGTCTCTCTGTGTCCCTATCTGTTACAGTCTCCTAAAGTATCCTCCTAAAGTCACCTCCTAACGTCCATCCTAAAGCCAACACCTAAAGCCTACACCTAACGACCCATCAAGTCAACGCCTATCTTAACGTTTAAACATAAAGACCAGACCTAAAGACCAGACCTAAAGACAC‑TACATCAACACCAAACCTAAAGACGCCTTGTTGTTACCCATAAATTGATAAACTTTAATCATTGTATTTATTAATa                                                                                                                                                                                               >  1:94984/1‑301 (MQ=255)`  
                                                                                                    `cccTAAGGGTTGGGGATGACCCTTGGGTTTGTCTTTTGGTCTTACCTTGAGTGTCTCTCTGTGTCCCTATCTGTTACAGTCTCCTAAAGTATCCTCCTAAAGTCACCTCCTAACGTCCATCCTAAAGCCAACACCTAAAGCCTACACCTAAATACCCATCAAGTCAACGCCTATCTTAACGTTTAAACATAAAGACCAGACCTAAAGACCAGACCTAAAGACAC‑TACATAAATACCAGACCTACAGACGCCTTGTTGTTAGCCATAAAGTGATAACCTTTAATCATTGTCTttattctgtc                                                                                                                                                                                              >  1:223338/1‑296 (MQ=255)`  
                                                                                                    `cccTAAGGGTTGGGGATGACCCTTGGGTTTGTCTTTGGGTGTTACCTTGAGTGTCTCTCTGTGTCCCTATCTGTTACAGTCTCCTAAAGTATCCTCCTAAAGTCACCTCCTAACGTCCATCCTAAAGCCAACACCTAAAGCCTACACCTAAAGACCCATCAAGTCAACGCCTATCTTAAAGTTTAAACATAAAGACCAGACCTAAAGACCAGACCTAAATACAC‑TACATAAAGACCAGACCTACAGACGCCTTGTTGTTAGCCATAAAGTGATAACCTTTAATCATTGTCTttattctgtc                                                                                                                                                                                              >  1:144086/1‑296 (MQ=255)`  
                                                                                                    `cccTAAGGGTTGGGGATGACCCTTGGGTTTGTCTTTGGGTGTTACCTTGAGTGTCTCTCTGTGTCCCTATCTGTTACAGTCTCCTAAAGTATCCTCCTAAAGTCACCTCCTAACGTCCATCCTAAAGCCAACACCTAAAGCCTACACCTAAAGACCCATCAAGTCAACGCCTATCTTAAAGTTTAAACATAAAGACCAGACCTAAAGACCAGACCTAAAGACAC‑TACATAAAGACCAGACCTAAAGACGCCTTGTTGTTAGCCATAAAGTGATAACCTTTAATCATTGTCTTTATTAATAc                                                                                                                                                                                              <  1:10439/301‑1 (MQ=255)`  
                                                                                                    `cccTAAGGGTTGGGGATGACCCTTGGGGTTGTCTTTGGGTGTTACCTTGAGTGTCTCTCTGTGTCCCTATCTGTTACAGTCTCCTAAAGTATCCTCCTAAAGTCACCTCCTAACGTCCATCCTAAAGCCAACACCTAAAGCCTACACCTAAAGACCCATCAAGTCAACGCCTATCTTAAAGTTTAAACATAAAGACCAGACCTAAAGACCAGACCTAAAGACAC‑TACATAAAGACCAGACCTAAAGACGCCTTGTTGTTAGCCATAAAGTGATAACCTTTAATCATTGTCTTTATTAATAc                                                                                                                                                                                              <  1:418343/301‑1 (MQ=255)`  
                                                                                                    `cccGAACGGTTGGGGATGACCCTTGGGTTTGTCTTTGGGTGTTACCTTGAGTGTCTCTCTGTGTCCCTATCTGTTACAGTCTCATAAAGTATCCTCCTAAAGTCACCTCCTAACGTCCATCCTAAAGCCAACACCTAAAGCCTACACCTAAAGACCAATCAAGTCAACGCCTATCTTAAAGTTTAAACATAAAGACCAGACCTAAAGACCAGACCTAAAGACAC‑TACATAAAGACCAGACCTAAAGACGCCTTGTTGTTAGCCATAAAGTGATAACCTTTAATCATTGTCTTTATTAATAc                                                                                                                                                                                              <  1:134439/301‑1 (MQ=255)`  
                                                                                                       `aaaGGGTTGGGGATGACCCTTGGGTTTGTCTTTGGGTGTTACCTTGAGTGTCTCTCTGTGTCCCTATCTGTTACAGTCTCCTAAAGTATCCTCATAAAGTCACCTCCTAACGTCCATCCTAAAGCCAACACCTAAAGCCTACACCTAAAGACCCATCAAGTCAACGCCTATCTTAAAGTTTAAACATAAAGACCAGACCTAAAGACCAGACCTAAAGACAC‑TACATAAAGACCAGACCTAAAGACGCCTTGTTGTTAGCCATAAAGTGATAACCTTTAATCATTGTCTTTATTAATACAAc                                                                                                                                                                                           <  1:454632/300‑1 (MQ=255)`  
                                                                                                          `gggTTGGGGATGACCCTTGGGTTTGTCTTTGGGTGTTACCTTGAGTGTCTCTCTGTGTCCCTATCTGTTACAGTCTCCTAAAGTATCCTCCTAAAGTCACCTCCTAACGTCCATCCTAAAGCCAACACCTAAAGCCTACACCTAAAGACCCATCAAGTCAACGCCTATCTTAAAGTTTAAACATAAAGACCAGACCTAAAGACCAGACCTAAAGACAC‑TACATAAAGACCAGACCTAAAGACGCCTTGTTGTTAGCCATAAAGTGATAACCTTTAATCATTGTCTTTTTTATTACatgcct                                                                                                                                                                                        >  1:115252/1‑296 (MQ=255)`  
                                                                                                                  `tgtgtataagagacagtgcTTTGGGTGTTACCTTGAGTGTCTCTCTGTGTCCCTATCTGTTACAGTCTCATAAAGTATCCTCCTAAAGTAACCTCCTAACGTCCATCCTAAAGCCAACACCTAAAGCATACACCTAAAGACCCATCAAGTCAACGCCTATCTTAAAGTTTAAACATAAAGACCAGACCTAAAGACCAGACCTAAAGACAC‑TACATAAAGACCAGACCTAAAGACGCCTTGTTGTTAGCCATAAAGTGATAACCTTTAATCATTGTCTTTATTAATACAACTCACTATAAgg                                                                                                                                                                                <  1:468604/283‑1 (MQ=255)`  
                                                                                                                  `tgtgtataagagacaggTCTTTGGGTGTTACCTTGAGTGTCTCTCTGTGTCCCTATCTGTTACAGTCTCCTAAAGTATCAGCCTAAAGTCACCTCCTAACGTCCATCCTAAAGCCAACACCTAAAGCCTACACCTAAAGACCCATCAAGTCAACGCCTATCTTAAAGTTTAAACATAGAGACCAGACCTAAAGACCAGACCTAAAGACAC‑TACATAAAGACCAGACCTAAAGACGCCTTGTTGTTAGCCATAAAGTGATAACCTTTAATCATTGTCTTTATTAATACAACTCACTATAAgg                                                                                                                                                                                <  1:106842/285‑1 (MQ=255)`  
                                                                                                                  `gATGACCCTTGTGTTTGTCTTTGGGTGTTACCTTGAGTGTCTCTCTGTGTCCCTATCTGTTACAGTCTCCTAAAGTATCCTCCGAAAGTCACCTCCTAACGTCCATCCTAAAGCCAACACCTAAAGCCTACACCTAAAGACCCATCAAGTCAACGCCTATCTTAAAGTTTAAACATAAAGACCAGACCTAAAGACCAGACCTAAAGACAC‑TACATAAAGACCAGACCTAAAGACGCCTTGTTGTTAGCCATAAAGTGATAACCTTTAATCATTGTCTTTATTAATACAACTCACTATAAgg                                                                                                                                                                                <  1:245810/301‑1 (MQ=255)`  
                                                                                                                  `gATGACCCTTGGGTTTGTCTTTGGGTGTTACCTTGAGTGTCTCTCTGTGTCCCTATCTGTTACAGTCTACTAAAGTATCCTCCTAAAGTCACCTCCTAACGTCCATCCTAAAGCCAACACCTAAAGCCTACACCTAAATACCCATCAAGTCAACGCCTATCTTAAAGTTTAAACATAAAGACCAGACCTAAAGACCAGACCTAAAGACAC‑TACATAAAGACCAGACCTAAAGACGCCTTGTTGTTAGCCATAAAGTGATAACCTTTAATCATTGTCTTTATTAATACAACTCACTATAAgg                                                                                                                                                                                <  1:348125/301‑1 (MQ=255)`  
                                                                                                                   `aTGACCCTTGGGTTTATCTTTGGGTTTTACCTTGAGTGTCTCACTGTGTCCCTATCTGTTACAGTCTCCTAAAGTATCCTCCGAAAGTCACCTCCTAACGTCCATCCTAAAGCCAACACCTAAAGCCTACACCTAAAAACCCATCAAGTCAACGCCTATCTTAAAGTTTAAACATAAAGACCAAACCTAAAGACCAGACCTAAAGACAC‑TACATAAAGACCAGACCTAAAGACGCCTTGTTGTTAGCCATAAAGTGATAACCTTTAATCATTGTCTTTATTAATACAACTCACTATAAGga                                                                                                                                                                               <  1:166154/301‑1 (MQ=255)`  
                                                                                                                       `cccTTTGGTTTGTCTTTGGGTGTTACCTTGAGTGTCTCTCTGTGTCCCTATCTGTTACAGTCTCCTAAAGTATCCTCCTAAAGTCACCTCCTAACGTCCATCCTAAAGCCAACACCTAAAGCCTACACCTAAAGACCCATCAAGTCAACGCCTATCTTAAAGTTTAAACATAAAGACCAGACCTAAAGACCAGACCTCAAGACAC‑TACATAAAGACCAGACCTAAAGACGCCTTGTTGTTAGCCATAAAGTTATAACCTTTAATCATTGTCTTTATTAATACAACTCACTATAAGaagaga                                                                                                                                                                           >  1:174186/1‑301 (MQ=255)`  
                                                                                                                       `cccTTGGGTTTGTCTTTGGGTGTTACCTTGAGTGTCTCTCTGTGTCCCTATCTGTTACAGTCTCCTAAAGTATCCTCCTAAAGTCACCTCCTAACGTCCATCCTAAAGCCAACACCTAAAGCCTACACCTAAAGACCCATCAAGTCAACGCCTATCTTAAAGTTTAAACATAAAGACCAGACCTAAAGACCAGACCTAAAGACAC‑TACATAAAGACCAGACCTAAAAACGCCTTGTTGTTAGCCATAAAGTGATAACCTTTAATCATTGTCTttattctttatcttatacaaatctccgga                                                                                                                                                                           >  1:371371/1‑277 (MQ=255)`  
                                                                                                                                  `gTCTTTGGGTGTTACCTTGAGTGTCTCTCTGTGTCCCTATCTGTTACAGTCTCCTAAAGTATCCTCCTAAAGTCACCTCCTAACGTCCATCCTAAAGCCAACACCTAAAGCCTACACCTAAAGACCCATCAAGTCGACGCCTATCTTAAAGTTTAAACATAAAGACCAGACCTAAAGACCAGACCTAAAGACAC‑TACATAAAGACCAGACCTAAAGACGCCTTGTTGTTAGCCATAAAATGATAACCTTTAATCATTGTCTTTATTAATACAACTCACTATAAGGAGAGACAACTTAAaga                                                                                                                                                                >  1:191230/1‑301 (MQ=255)`  
                                                                                                                                          `gtgtTACCTTGAGTGTCTCTCTGTGTCCCTATCTGTTACAGTCTCCTAAAGTATCCTCCTAAAGTCACCTCCTAACGTCCATCCTAAAGTCAACACCTAAAGCCTACACCTAAAGACCCATCAAGTCAACGCCTATCTTAAAGTTTAAACATAAAGACCAGACCTAACGACCAGACCTAAAGACAC‑TACATAAAGACCAGACCTAAAGACGCCTTGTTGTTACCCATAAAGTGATAACCTTTAATCATTTTCTTTATTACTACAACTCCCTATAAgtatctgtctcttatacacatctcct                                                                                                                                                        >  1:161291/1‑276 (MQ=255)`  
                                                                                                                                           `aagagacaggAGTGTCTCTCTGTGTCCCTATCTGTTACAGTCTCCTAAAGTATCCTCCTAAAGTCACCTCCTAACGTCCATCCTAAAGCCAACACCTAAAGCCTACACCTAAAGACCCATCAAGTCAACGCCTATCTTAAAGTTTAAACATAAAGACCAGACCTAAAGACCAGACCTAAAGACAC‑TACATAAAGACCAGACCTAAAGACGCCTTGTTGTTAGCCATAAAGTGATAACCTTTAATCATTGTCTTTATTAATACAACTCACTATAAGGAGAGACAACTTAAAGAGACTTaaaa                                                                                                                                                       <  1:183018/292‑1 (MQ=255)`  
                                                                                                                                                          `ctctctGTGTCCCTATCTGTTACAGTCTCCTAAAGTATCCTCCTAAAGTCACCTCCTAACGTCCATCCTAAAGCCAACACCTAAAGCCTACACCTAAAGACCCATCAAGTCAACGCCTATCTTAAAGTTTAAACATAAAGACCAGACCTAAAGACCAGACCTAAAGACAC‑TACATAAAGACCAGACCTAAAGACGCCTTGTTTTTAGCCATAAAGTGATAACCTTTAATCATTGTCTTTATTAATACAACCAACTATAAGGATAAAAAACTTAAAGAGACTTAAAAGATTAATTTAAAAtt                                                                                                                                        >  1:318189/1‑301 (MQ=255)`  
                                                                                                                                                              `ctGTGTCCCTATCTGTTACAGTCTCCTAAAGTATCCTCCTAAAGTCACCTCCTAACGTCCATCCTAAAGCCAACACCTAAAGCCTACACCTAACGACCCATCAAGTCAACGCCTATCTTAAAGTTTAACCATAAAGACCAGACCTAAAGACCAGACCTAAAGACAC‑TACATAAAGACCAGACCTAAAGACGCCTTGTTGTTAGCCATAAAGTGATAACCTTTAATCATTGTATTTATTAATACAACTCACAATAAGGAGAGACACCTTAAAGAGACTTAAAAGATTAATATaaaactgtat                                                                                                                                    >  1:34435/1‑295 (MQ=255)`  
                                                                                                                                                              `ctGTGTCCCTATCTGTTACAGTCTCCTAAAGTATCCTCCTAAAGTCACCTCCTAACGTCCATCCTAAAGCCAACACCTAAAGCCTACACCTAAAGACCCATCAAGTCAACGCCTATCTTAAAGTTTAAACATAAAGACCAGACCCAAAGACCAGACCTAAAGACAC‑TACATAAAGACCAGACCTAAAGACGCCTTGTTGTTAGCCATAAAGTGATAACCTTTAATCATTGTCTTTATTAATACAACTCACTATAAGGAAAGACAACTTAAagagctgtctcttatacacatctccgagccc                                                                                                                                    >  1:27365/1‑274 (MQ=255)`  
                                                                                                                                                                  `gtCCCTATCTGTTACAGTCTCCTAAAGTATCCTCCTAAAGTCACCTCCTAACGTCCATCCTAAATCCAACACCTAAAGCCTACACCTAAAGACCCATCAAGTCAACGCCTATCTTAAAGTTTAAACATAAAGACCAGACCTAAAGACCAGACCTAAAGACAC‑TACATAAAGACCAGACCTAAAGACGCCTTGTTGTTAGCCATAAAGTGATAACCTTTAATCATTGTCTTTATTAATACAACTCACTATAAGGAGAGACAACTTAAAGAGACTTAAAAGATTAATTTAAAATTTATCaaaa                                                                                                                                >  1:376447/1‑301 (MQ=255)`  
                                                                                                                                                                  `gtCCCTATCTGTTACAGTCTCCTAAAGTATCCTCCTAAAGTCACCTCCTAACGTCCATCCTAAAGCCAACACCTAAAGCCTACACCTAAAGACCCATCAAGTCAACGCCTATCTTAAAGTTTAAACATAAAGACCAGACCTAAAGACCAGACCTAAAGACAC‑TACATAAAGACCAGACCTAAAGACGCCTTGTTGTTAGCCATAAAGTGATAACCTTTAATCATTGTCTTTCTTAATACAACTCACTATAAGGAGAGAAAACTTAAAGAGACTTAAAAAATTAATTTAAAATTTATCaaaa                                                                                                                                >  1:77263/1‑301 (MQ=255)`  
                                                                                                                                                                  `gtCCCTATCTGTTACAGTCTCCTAAAGTATCCTCCTAAAGTCACCTCCTAACGTCCATCCTAAAGCCAACACCTAAAGCCTACACCTAAAGACCCATCAAGTCAACGCCTATCTTAAAGTTTAAACATAAAGACCAGACCTAAAGACCAGACCTAAAGACAC‑TACATAAAGACCAGACCTAAAGACGCCTTGTTGTTAGCCATAAAGTGATAACCTTTAATCATTGTCTTTATTAATACAACTCACTATAAGGATAGACAACTTAAAGAGACTTAAAAGATTAATTTAAAATTTATCaaaa                                                                                                                                >  1:412102/1‑301 (MQ=255)`  
                                                                                                                                                                         `tCTGTTACAGTCTCCTAAAGTATCCTCCTAAAGTCACCTCCTAACGTCCATCCTAAAGCCAACACCTAAAGCCTACACCTAAAGACCCAACAAGTCAACGCCTATCTTAAAGTTTAAACATAAAGAACAGTCCTAAAGACCAGACCTAAAGACAC‑TACATAAAGACCAGACCTAAAGACGCCTTGTTGTTAGCCATAAAGTGATAACCTTTAATCATTGTCTTTATTAATACAACTCACTATAAGGAGAGACAACTTAAAGAGACTTAAAAGATTAATTTAAAATTTATCAAAAAGAGTAt                                                                                                                         <  1:323255/301‑1 (MQ=255)`  
                                                                                                                                                                         `gcTGTTACAGTCTCCTAAAGTATCCTCCTAAAGTCACCTCCTAACGTCCATCATAAAGCCAACACCTAAAGCCTACACCTAAAGACCCATCAAGTCAACGCCTATCTTAAAGTTTAAACATAAAGACCAGACCTAAAGACCAGACCTAAAGACAC‑TACATAAAGACCAGACCTAAAGACGCCTTGTTGTTAGCCATAAAGTGATAACCTTTAATCATTGTCTTTATTAATACAACTCACTATAAGGAGAGACAACTTAAAGAGACTTAAAAGATTAATTTAAAATTTATAAAAAAGAGTAt                                                                                                                         <  1:464121/300‑1 (MQ=255)`  
                                                                                                                                                                                  `gTCTCCTAAAGTATCCTCCTAAAGTCACCTCCTAACGTCCATCCTAAAGCCAACACCTAAAGCCTACACCTAAAGACCCATCAAGTCAACGCCTATCTTAAAGTTTAAACATACAGACCAGACCTAAAGACCACACCTAAAGACAC‑TACATAAAGACCAGACCTAAAGAAGCCTTGTTGTTAGCCATAAAGTGCTAACCTTTAATCATTGTCTTTATTAATACACCTCCCTATAAGGAGACACAACTTAAAGATACTTCAACGATTAATTTAAAATTTATCAAAAAGAGTATTGACTTaaa                                                                                                                >  1:504733/1‑301 (MQ=255)`  
                                                                                                                                                                                  `gTCTCCTAAAGTATCCTCCTAAAGTCACCTCCTAACGTCCATCCTAAAGCCAACACCTAAAGCCTACACCTAAAGACCCATCAAGTCAACGCCTATCTTAAAGTTTAAACATAAAGACCAGACCTAAAGACCAGACCTAAAGACAC‑TACATAAAGACCAGACCTAAAGACGCCTTGTTGTTAGCCATAAAGTGATAACCTTTAATCATTGTCTTTATTAATACAACTCACTATAAGGAGAGACAACTTAAAGAGACTTAAAAGATTAATTTAAAATTTATCAAAAAGAGTATTGACTTaaa                                                                                                                >  1:186989/1‑301 (MQ=255)`  
                                                                                                                                                                                  `gTCTCCTAAAGTATCCTCCTAAAGTCACCTCCTAACGTCCATCCTAAAGCCAACACCTAAAGCCTACACCTAAAGACCCATCAAGTCAACGCCTATCTTAAAGTTTAAACATAAAGACCAGACCTAAAGACCAGACCTAAAGACAC‑TACATAAAGACAAGACCTAAAGACGCCTTGTTGTTAGCCATAAAGTGATAACCTTTAATCATTGTCTTTATTAATACAACTCACTATAAGGAGAGACAACTTAAAGAGACTTAAAAGATTAATTTAAAATTTATCAAAAAGAGTATTGACTTaaa                                                                                                                >  1:228556/1‑301 (MQ=255)`  
                                                                                                                                                                                   `gtgggctcggagatttgtataagagacagcctAACGTCCATCCTAAAGCCAACACCTAAAGCCTACACCTAAAGACCCATCAAGTCAACGCCTATCTTAAAGTTTAAACATAAAGACCAGACCTAAAGACCAGACCTAAAGACAC‑TACATAAAGACCAGACCTAAAGACGCCTTGTTGTTAGCCATAAAGTGATAACCTTTAATCATTGTCTTTATTAATACAACTCACTATAAGGAGAGACAACTTAAAGAGACTTAAAAGATTAATTTAAAATTTATCAAAAAGAGTATTGACTTAAAg                                                                                                               <  1:252670/272‑1 (MQ=255)`  
                                                                                                                                                                                      `ccTAAAGTATCCTCCTAAAGTCACCTCCTAACGTCCATCCTAAAGCCAAAACCTAAAGCCTACACCTAAAGACCCATCAAGTCAACGCCTATCTTAAAGTTTAAACATAAAGACCAGACCTAAAGACCAGACCTAAAGACAC‑TACATAAAAACCAGACCTAAAGACGCCTTGTTGTTAGCCATAACGTGTTAACATTTAATCATTTTCTTTTTTAATACAACTTACTATTTGGAGAGACGACTTAAAGAGACTTAAAAAATTAATTTACAATTTATTAAAACAAGTATTGAATCAAAGTCt                                                                                                            >  1:82818/1‑301 (MQ=255)`  
                                                                                                                                                                                             `acacctcctAAAGTCACCTCCTAACGGCCATCCTAAAGCCAACACCTACAGCCTACACCTAAAGACCCATCAAGTCAACGCCTATCTTAAAGTTTAAACATAAAGACCAGAACTAAAGACCATACCTAAAGACAC‑TACAGAAAGACCAGACCTAAAGACTCCTTGTTGTTAGCCATAAAGTGATAACCTTTAATCAGTGTCTTTATTAATACAACTCACTATAAGGAGAGACAACTTAAAGAGACTTAAAAGATTAATTTAAAATTTATCAAAAAGAGTATTGACTTAAAGTCTAACCtat                                                                                                     <  1:489741/298‑1 (MQ=255)`  
                                                                                                                                                                                              `aTCCTCCTAAAGTCACCCCCTAACGTCCATCCTAAAGCCAACACCTAAAGCCTACACCTAAAGACACATCAAGTCAACGCCTATCTTAAAGTTTAAACAT‑AAGACCAGACCTAAAGACCAGACCTAAAGACAC‑TACATAAAGACCAGACCTAAAGACGCCTTGTTGTTAGCCATAAAGTGATAACCTTTAATCATTGTCTTTATTAATACAACTCACTATAAGGAGAGACAACTTAAAGAGACTTAAAAGATTAATTTAAAATTTATCAAAAAGAGTATTGACTTAAAGTCTAACCTATAg                                                                                                   <  1:398944/301‑1 (MQ=255)`  
                                                                                                                                                                                                `cctcctAAAGTCACCTCCTAACGTCCATCCTAAAGCCAACACCTAAAGCCTACACCTAAAGACCCATCAAGTCAACGCCTATCTTAAAGTTTAAACATAAAGACCAGACCTAAAGACCAGACCTAAAGACAC‑TACATAAAGACCAGACCTAAAGACGCCTTGTTGTTAGCCATAAAGTGATAACCTTTAATCATTGTCTTTATTAATACAACTCACTATAAGGAGAGACAACTTAAAGAGACTTAAAAGATTAATTTAAAATTTATCAAAAAGAGTATTGACTTAAAGTCTAACCTATAgg                                                                                                  >  1:362815/1‑301 (MQ=255)`  
                                                                                                                                                                                                 `ctcctAAAGTCACCTCCTAACGTCCATCCTAAAGCCAACACCTAAAGCCTACACCTAAAGACCCATTAAGTCAACGCCTATCTTAAAGTTTAAACATAAAGACCAGACCTAAAGACCAGACCTAAAGACAC‑TACATAAAGACCAGATCTAAAGACGCCTTGTTGTTAGATATAAAGTGATAACCTTTAATCATTGTCTTTATTAATACAACTCACTATAAGGAGAGACAACTTAAAATGACTTAAAAGACTAATTAAAAATTTATCAACAAGCGTATTGACTTAAAGTTTatcttttagaa                                                                                                 >  1:352351/1‑291 (MQ=255)`  
                                                                                                                                                                                                 `ctcctAAAGTCACCTCCTAACGTCCATCCTAAAGCCAACACCTAAAGCCTACACCTAAAGACCCATCAAGTCAACGCCTATCTTAAAGTTTAAACATCAAGACCAGACCTAAAGACCAGACCTAAAGACAC‑TACATAAAGACCAGACCTAAAGACGCCTTGTTGTTAGCCATCAAGTGATAACCTTTAATCATTGTCTTTATTAATACAACTCACTATAAGGAGATACACCTTAAAGAGACTTAACAGATTAATTTAAAATTTATCAAAGAGAGTATTGACTTAAAGTCTAACCTATAgtc                                                                                                 >  1:402647/1‑299 (MQ=255)`  
                                                                                                                                                                                                  `tcctAAAGTCACCTCCGAACGTCCATCCTAAAGCCAACACCTAAAGCCTACACCTAAAGACCCATCAAGTCAACGCCTATCTTAAAGTTTAAACATAAAGACCAGACCTAAAGACCAGACCTAAAGACAC‑TAAATAAAGACCAGACCTAAAGACGCCTTGTTGTTAGCCATAAAGTGATAACCTTTAATCATTGTCTTTATTAATACAACTCACTATAAGGAGAGACAACTTAAAGAGACTTAAAAGATTAATGTAAAATTTATCAAAAAGAGTATTGACTTAAAGTCTAACCTATAGGAt                                                                                                <  1:303454/301‑1 (MQ=255)`  
                                                                                                                                                                                                      `aaaagcACCGCCTAACGTCCAACCTAAAGCCAACACCGAAAGCCTACACCTAAAAACCAATCAAGTCAACGCCTATCTTAAAGTTTAAACATAAAGACCAGACCTAAAGACCAGAACTAAAGACAC‑TACATAAAAACCAGACCTAAAGACGCCGTGTTGTTAGCCATAAAGTGATAACCTTTAATCATTGTCTTTATTAATACAACGCACTATAAGGAGAGACAACTTAAAGAGACTTAAAAGATTAATTTAAAATTTATCAAAAAGAGTATTGACTTAAAGTCTAACCTATAGGATACtt                                                                                            <  1:500454/296‑1 (MQ=255)`  
                                                                                                                                                                                                          `atgtgtataagagacagtCCTAAAGCCAACACCTAAAGCCTACACCTAAAGACCCATCAAGTCAACGCCTATCTTAAAGTTTAAACATAAAGACCAGACCTAAAGACCAGACCTAAAGACAC‑TACATAAAGACCAGACCTAAAGACGCCTTGTTGTTAGCCATAAAGTGATAACCTTTAATCATTGTCTTTATTAATACAACTCACTATAAGGAGAGACAACTTAAAGAGACTTAAAAGATTAATTTAAAATTTATCAAAAAGAGTATTGACTTAAAGTCTAACCTATAGGATACTTACAg                                                                                        <  1:445492/284‑1 (MQ=255)`  
                                                                                                                                                                                                             `cctcctAACGTCCATCCTAAAGCCAACACCTAAAGCCTACACCTAAAGACCCATCAAGTCAACGCCTATCTTAAAGTTTAAACATAAAGACCAGACCTAAAGACCAGACCTAAAAACAC‑TACACAAAGACCAGACCTAAAGACGCCTTGTTGTTAGCCATAAAGTGACAACCTTTAATCATTGTCTTTATTAATACAACTCACTATAATGAGAGACAACTTAAAGAGACTTAAAAGATTAATTTAAAATTTATCAAAATGAGTATTTACTTAAAGTCTAACCTATAGGATAATTACAGCCa                                                                                     >  1:340099/1‑301 (MQ=255)`  
                                                                                                                                                                                                             `cctcctAACGTCCATCCTAAAGCCAACACCTAAAGCCTACACCTAAAGACCCATCAAGTCAACGCCTATCTTAAAGTTTAAACATAAAGACCAGACCTAAAGACCAGACATAAAGACAC‑TACATAAAGACAAGACCTAAAGACGCCTTGTTGTTAGCCATAAAGTGATAACCTTTAATCATTGTCTTTATTAATACAACTCACTATAAGGAGAGACAACTTAAAGAGACTTAAAAGATTAATTTAAAATTTATCAAAAAGAGTATTGACTTAAAGTCTAACCTATAGGATACTTACATcct                                                                                     >  1:285352/1‑300 (MQ=255)`  
                                                                                                                                                                                                                `cctAACGTCTATCCTAAAGCCAACAACGAAATCCAAAACCTAAAGACCCAACAAGTCAACGCCTATCTTAAAGTTTAAACATAAAGACCATACCTAAAGACCAGACCTAAAGACAC‑TACATAAAGACCAGACCTAAAGACGCCTTGTTGTTAGCCATAAAGTGATAACCTTTATTCATTGTATTTATTAATACAAATCACTATAAGTAGAGACAACTTAAAGAGACTTAAAAGATTAATTTAAAATTTATCAAAAAGAGTATTGACTTAAAGTCTAACCTATAGGATACTTACAGCCATCg                                                                                  <  1:55832/301‑1 (MQ=255)`  
                                                                                                                                                                                                                  `tAACGTCCATCCTAAAGCCAACACCTAAAGCCTACACCTAAAGACCCATCAAGTCAACGCCTATCTTAAAGTTTAAACATAAAGACCAGACCTAAAGACCAGACCTAAAGACAC‑TACATAAAGACCAGACCTAAAGACGCCTTGTTGTTAGCCATAAAGTGATAACCTTTAATCATTGTCTTTATTAATACAACTCACTATAAGGAGAGACAACTTAAAGAGACTTAAAAGATTAATTTAAAATTTATCAAAAAGAGTATTGACTTAAAGTCTAACCTATAGGATACTTACAGCCATCgag                                                                                <  1:405763/301‑1 (MQ=255)`  
                                                                                                                                                                                                                     `cGTCCATCCTAAAGCCAACACCTAAAGCCTACACCTAAAGACCCATCAAGTCAACGCCTATCTTAAAGTTTAAACATAAAGACCAGACCTAAAGACCAGACCTAAAGACAC‑TACATAAAGATCAGACCTAAAGACACCTTGTTGTTATCCATAAAGTGATAACCTTTAATCATTGTCTTTATTAATACAACTCACTATAAGGAGAGACAACTTAAAGAGACTTAAAAGATTAATTTAAAATTTATCAAAAAGAGTATTGACTTAAAGTCTAACCTATAGGATACTTACAGCCATCGAGAgg                                                                             >  1:394233/1‑301 (MQ=255)`  
                                                                                                                                                                                                                        `aagtCCTAAAGCCAACACCTAAAGCCTACACCTAAAGACCCATCAAGTCAACGCCTATCTTAAAGTTTAAACATAAAGACCAGACCTAAATACCAGACCTAAGGACAC‑TACATAAAGACCAGACCTAAAGACGCCTTTTTGTTAGCCATAAAGTGATAACCTTTAATCATTGTCTTTATTAATACAACTCACTATAAGGAGAGACAACTTAAAGAGACTTAAAAGATTAATTTAAAATTTATCAAAAAGAGTATTGACTTAAAGTCTAACCTATAGGATACTTACAGCCATCGAGAGGGac                                                                          <  1:61175/298‑1 (MQ=255)`  
                                                                                                                                                                                                                                `aaGCCAACACCTAAAGCCTACACCTAAAGACCCATCAAGTCAACGCCTATCTTAAAGTTTAAACATAAAGACCAGACCTAAAGACCAGACCTAAAGACAC‑TACATAAAGACCAGACCTAAAGACGCCTTGTTGTTAGCCATAAAGTGATAACCTTTAATCATTGTCATTATTAATACAACTCACTATAAGAAGAGACAACTTAAAGAGAATTAAAAGATTAATTTAAAATTTATCAAAAAAAGTATTAACTTAAAGTCTAACCTATAGGATACTGACAAACATCGAGACGGacatgtctct                                                                  >  1:425638/1‑294 (MQ=255)`  
                                                                                                                                                                                                                                 `ggagatgtgtataagagacagccTAAAGACCCATCAAGTCAACGCCTATCTTAAAGTTTAAACATAAAGACCAGACCTAAAGACCAGACCTAAAGACAC‑TACATAAAGACCAGACCTAAAGACGCCTTGTTGTTAGCCATAAAGTGATAACCTTTAATCATTGTCTTTATTAATACAACTCACTATAAGGAGAGACAACTTAAAGAGACTTAAAAGATTAATTTAAAATTTATCAAAAAGAGTATTGACTTAAAGTCTAACCTATAGGATACTTACAGCCATCGAGAGGGACACGGCGAAt                                                                 <  1:129764/280‑1 (MQ=255)`  
                                                                                                                                                                                                                                 `agagacagccTAAAGCCTACACCTAAAGACCCAGCAAGTCAAAGCCTATCTTAAAGTTTAAACATAAAGACCAGACCTAAAGACCAGACCTAAAGACAC‑TACATAAAGACCAGACCTAAAGACGCCTTGTTGTTAGCCATAAAGTGATAACCTTTAATCATTGTCTTTATTAATACAACTCACTATAAGGAGAGACAACTTAAAGAGACTTAAAGGATTAATTTAAAATTTATCAAAAAGAGTATTGACTTAAAGTCTAACCTATAGGATACTTACAGCCATCGAGAGGGACACGGCGAAt                                                                 <  1:307600/293‑1 (MQ=255)`  
                                                                                                                                                                                                                                    `cAACACCTAAAGCCTACACCAAAAGACCCATCAAGTAAACGCCTATCTTAAAGTTTAAACATAAAGACCAGACCTAAAGACCAGACCTAAATACAC‑TACATAAAGACCAGACCTAAAGACGCCATTTTGTTAGCCATAAAGTGATAACCTTGAATCATTGTCTTTATTAATACAACTCACTATAATGAGAGACAACTTAAAGAGACTTAAAAGATTAATTTAAAATTTATCAAAAAGAGTATTGACTTAAAGTCTAACCTATAGGATACTGACAGCCATCGAGAGGGACACGGCGAATAGc                                                              <  1:110926/301‑1 (MQ=255)`  
                                                                                                                                                                                                                                         `ccTACAGACTACACCTAAAGACCCATCAAATCTACGCCTATCTTAAAGTTTAAACATAAAGACCAGACCTAAAGACCAGACCTAAAGACAC‑TACATAAAGACCAGACCTAAAGACGCCTTGTTGTTAGCCATAAAGTGATAACCTTTAATCATTGTCTTTATTAATACAACTCACTATAAGGAGAGACAACTTAAAGAGACTTAAAAGATTAATTTAAAATTTATCAAAAAGAGTATTGACTTAAAGTCTAACCTATAGGATACTTACAGCCATCGAGAGGGACACGGCGAATAGCCATcc                                                         <  1:156689/301‑1 (MQ=255)`  
                                                                                                                                                                                                                                          `cTAAAGCCTACACCTAAAGACCCATCAAGTCAACGCCTATCTTAAAGTTTAAACATAAAGACCAGACCTAAAGACCAGACCTAAAGACAC‑TACATAAAGACCAGACCTAAAGACGCCTTGTTGTTAGCCATAAAGTGATAACCTTTAATCATTGTCTTTATTAATACAACTCACTATAAGGAGAGACAACTTAAAGAGACTTAAAAGATTAATTTAAAATTTATCAAAAAGAGTATTGACTTAAAGTCTAACCTATAGGATACTTACAGCCATCGAGAGGGACACGGCGAATAGCCATccc                                                        <  1:237742/301‑1 (MQ=255)`  
                                                                                                                                                                                                                                                  `acagacTAAAGACCCATCAAGTCAACGCCTATCTTAAAGTTTAAACATAAAGACCATACCTAAAGACCAGACCTAAAGACAC‑TACATAAAGACCAGACCTAAAGACGCCTTGTTGTTAGCCATAAAGTGATAACCTTTAATCATTGTCTTTATTAATACAACTCACTATAAGGAGAGACAACTTAAAGAGACTTAAAAGATTAATTTAAAATTTATCAAAAAGAGTATTTACTTAAAGTCTAACCTATAGGATACTCACAGCCATCGAGAGGGACACGGCgtccctctcgatggctgtaag                                                <  1:147404/296‑21 (MQ=255)`  
                                                                                                                                                                                                                                                    `cacCTAAAGACCCATCAAGTCAACGCCTATCTTAAAGTTTAAACATAAAGACCAGACCTAAAGACCAGACCTAAAGACAC‑TACATAAAGACCAGACCTAAAGACGCCTTGTTGTTAGCCATAAAGTGATAACCTTTAATCATTGTCTTTATTAATAAAACTCACTATAAGGAGAGACAACTTAAAGAGACTTAAAAGATTAATTTAAAATTTATCAAAAAGAGTATTGAGTTAAAGTCTAACGTATAGGTTACTTAAAGCCATCGAGGGGGACACGGCGAATAACCATCCCAATCGACAcc                                              >  1:100062/1‑301 (MQ=255)`  
                                                                                                                                                                                                                                                      `ccTAAAGACCCATCAAGTCAACGCCTATCTTAAAGTTTAAACATAAAGACCAGACCTAAAGACCAGACCTAAAGACAC‑TACATAAAGACCAGACCTAAAGACGCCTTGTTGTTAGCCATAAAGTGATAACCTTTAATCATTGTCTTTATTAATACAACTCACTATAAGGAGAGACAACTTAAAGAGACTTAAAAGATTAATTTAAAATTTATCAAAAAGAGTATTGACTTAAAGTCTAACCTATAGGATACTTACAGCCATCTAGAGGGACACGGCGAATAGCCATCCCAATCGACACCgg                                            >  1:86796/1‑301 (MQ=255)`  
                                                                                                                                                                                                                                                              `cccATCAAGTCAACGCCTATCTTAAAGTTTAAACATAAAGACCAGACCTAAAGACCAGACCTAAAGACAC‑TACATAAAGACCAGACCTAAAGACGCCTTGTTGTTAGCCATAAAGTGATAACCTTTAATCATTGTCTTTATTAATACAACTCACTATAAGGAGAGACAACTTAAAGAGACTTAAAAGATTAATTTAAAATTTATCAAAAAGAGTATTGACTTAAAGTCTAACCTATAGGATACTTACAGCCATCGAGAGGGACACGGCGAATAGCCATCCCAATCGACACCGGGGTctgtc                                    >  1:477069/1‑297 (MQ=255)`  
                                                                                                                                                                                                                                                              `cccATCAAGTCAACGCCTATCTTAAAGTTTAAACATAAAGACCAGACCTAAAGACCAGACCTAAAGACAC‑TACATAAAGACCAGACCTAAAGACGCCTTGTTGTTAGCCATAAAGTGATAACCTTTAATCATTGTCTTTATTAATACAACTCACTATAAGGAGAGACAACTTAAAGAGACTTAAAAGATTAATTTAAAATTTATCAAAAAGAGTATTGACTTAAAGTCTAACCTATAGGATACATACAGCCATAGAGAGGGACACGGCGAAAAGCCAGCGCAATCGACACAGGGGTCAAcc                                    >  1:66450/1‑301 (MQ=255)`  
                                                                                                                                                                                                                                                              `cccATCAAGTCAACGCCTATCTTAAAGTTTAAACATAAAGACCAGACCTAAAGACCAGACCTAAAGACAC‑TACATAAAGACCAGACCTAAAGACGCCTTGTTGTTAGCCATAAAGTGATAACCTTTAATAATTGTCTTTATTAATACAACTCACTATAAGGAGAGACAACTTAAAGAGACTTAAAAGATTAATTTAAAATTTATCAAAAAGAGTATTGACTTAAAGTCTAACCTATAGGATACTTACAGCCATCGAGAGGGACACGGCGAATAGCCATCCCAATCGACACCGGGGTCAAcc                                    >  1:169322/1‑301 (MQ=255)`  
                                                                                                                                                                                                                                                                     `aGTCAACGCCTATCTTAAAGTTTAAACATAAAGACCAGACCTAAAGACCAGACCTAAAGACAC‑TACATAAAGACCAGACCTAAAGACGCCTTGTTGTTAGCCATAAAGTGATAACCTTTAATCATTGTCTTTATTAATACAACTCACTATAAGGAGAGACAACTTAAAGAGACTTAAAAGATTAATTTAAAATTTATCAAAAAGAGTATTGACTTAAAGTCTAACCTATAGGATACTTACAGCCATCGAGAGGGACACGGCGAATAGCCATCCCAATCGACACCGGGGTCAACCGGATAAg                             <  1:290028/301‑1 (MQ=255)`  
                                                                                                                                                                                                                                                                       `acAACGCCTATCTGAAAGTTTAAACATAAAGACCAGACCTAAAGACCAGAACTAAAGACAC‑TACATAAAGACCAGACCTAAAGACGCCTTGTTGTTAGCCATAAAGTGATAACCTTTAATCAGTGTCTTTATTAATACAACTCACTATAAGGAGAGACAACGTAAAGAGACTTAAAAGATTAATTTAAAATTTATCAAAAAGAGTATTGACTTAAAGTCTAACCTATAGGATACTTACAGCCATCGAGAGGGACACGGCGAATAGCCATCCCAATCGACACCGGGGTCAACCGGATAAGTa                           <  1:332524/300‑1 (MQ=255)`  
                                                                                                                                                                                                                                                                        `tttgtagaagatacaggTTTAAACATAAAGACCAGACCGTACGACCCGACCTAAAGTAAC‑TACATAAATACCCGACCTAAAGATGCCTTGTTGTTAGCCATAAAGTGATAACCTTTAATCATTGTCTTTATTAATACAACTCACTATAAGGAGAGAAAACTTAAATAGACTTAATAGATTAATTTAAAATTTATCAAAAAGAGTATTGCCTTAAAGTCTAACCTATAGGATACTTACAGCCATCGAGAGTGACACGGCGAATAGCCATCCCAATCGACACCGGAGTCAACCGGATAAGTat                          <  1:51226/285‑2 (MQ=255)`  
                                                                                                                                                                                                                                                                        `aaaCGCCTATCTTAAAGTTTAAACATAAAGACCAGACCTAAAGACCAGACCTAAAGACAC‑TACATAAAGACCAGACCTAAAGACGCCTTGTTGTTAGCCATAAAGTGATAACCTTTAATCATTGTCTTTATTAATACAACTCACTATAAGGAGAGACAACTTAAAGAGACTTAAAAGATTAATTTAAAATTTATCAAAAAGAGTATTGACTTAAAGTCTAACCTATAGGATACTTACAGCCATCGAGAGGGACACGGCGAATAGCCATCCCAATCGACACCGGGGTCAACCGGATAAGTAg                          <  1:178546/300‑1 (MQ=255)`  
                                                                                                                                                                                                                                                                         `aaCGCCTATCTTAAAGTTTAAACATAAAGACCCGACCTAAAGA‑CAGACCTAAAGACAC‑TACATAAAGACCATACCTAAAGACGCCTTGTTGTTAGACATAAAGTGATAACCTTTAATCATTGTCTTTATTAATACAACTCACTATAAGGAGAGACAACTTAAAGAGACTTAAAAGATTAATTTAAAATTTATCAAAAAGAGTATTGACTTAAAGTCTAACCTATAGGATACTTACAGCCATCGAGAGGGACACGGCGAATAGCCATCCCAATCGACACCGGGGTCAACCGGATAAGTAGAc                        <  1:123731/301‑1 (MQ=255)`  
                                                                                                                                                                                                                                                                              `cTATATTAAAGTTTAAAAATAAAGACCAGACCTAAAGACCAGACCTAAAGACAC‑TACATAAAGACCAGACATAAAGACGCCTTGTTGTTAGCCATAAAGTGATAACCTTTAATCATTGTCTTTATTAATACAACTCACTATAAGGAGAGACAACTTAAAGAGACTTAAAAGATTAATTTAAAATTTATCAAAAAGAGTATTGACTTAAAGTCTAACCTATAGGATACTTACAGCCATCGAGAGGGACACGGCGAATAGCCATCCCAATCGACACCGGGGTCAACCGGATAAGTAGACAGcc                    <  1:281362/301‑1 (MQ=255)`  
                                                                                                                                                                                                                                                                                     `aaaagttAAACATAAAGACCAGACCTAAAGACCAGACCTAAAGACAC‑TACATAAAGACCAGACCTAAAGACGCCTTGTTGTTAGACATAAAGTGATAACCTTTAATCATTGTCTTTATTAATACAACTCACTATAAGGAGAGACAACTTAAAGAGACTTAAAAGATTAATTTAAAATTTATCAAAAAGAGTATTGACTTAAAGTCTAACCTATAGGATACTTACAGCCATCGAGAGGGACACGGCGAATAGCCATCCCAATCGACACCGGGGTCAACCGGATAAGTAGACAGCCTGATAAg             <  1:496218/296‑1 (MQ=255)`  
                                                                                                                                                                                                                                                                                               `cATAAAGACCAGACCTAAAGACCAGACCTAAAGACAC‑TACATAAAGACCAGACCTAAAGACGCCTTGTTGTTAGCCATAAAGTGATAACCTTTAATCATTGTCTTTATTAATACAACTCACTATAAGGAGAGACAACTTAAAGAGACTTAAAAGATTAATTTAAAATTTATCAAAAAGAGTATTGACTTAAAGTCTAACCTATAGGATACTTACAGCCATCGAGAGGGACACGGCGAATAGCCATCCCAATCGACACCGGGGTAAACCGGATAAGTAGACAGCCTGATAAGTCGCACGaaa   >  1:459265/1‑301 (MQ=255)`  

                                                                                                                                                                                                                                                                                                `|`                                                                                                                                                                                                                                                                                                               
`‑‑‑‑‑‑‑‑TCTCACAGTGTACGGACCTAAAGTTCCCCCATAGGGGGTACCTAAAGCCCAGCCAATCACCTAAAGTCAACCTTCGGTTGACCTTGAGGGTTCCCTAAGGGTTGGGGATGACCCTTGGGTTTGTCTTTGGGTGTTACCTTGAGTGTCTCTCTGTGTCCCTATCTGTTACAGTCTCCTAAAGTATCCTCCTAAAGTCACCTCCTAACGTCCATCCTAAAGCCAACACCTAAAGCCTACACCTAAAGACCCATCAAGTCAACGCCTATCTTAAAGTTTAAACATAAAGACCAGACCTAAAGACCAGACCTAAAGACAC‑TACATAAAGACCAGACCTAAAGACGCCTTGTTGTTAGCCATAAAGTGATAACCTTTAATCATTGTCTTTATTAATACAACTCACTATAAGGAGAGACAACTTAAAGAGACTTAAAAGATTAATTTAAAATTTATCAAAAAGAGTATTGACTTAAAGTCTAACCTATAGGATACTTACAGCCATCGAGAGGGACACGGCGAATAGCCATCCCAATCGACACCGGGGTCAACCGGATAAGTAGACAGCCTGATAAGTCGCACGAAAA  >  NC_001604.1/1‑581`

  

Alignment Legend

Aligned base mismatch/match (shaded by quality score): `ATCG/ATCG` `< 3 ≤` `ATCG/ATCG` `< 10 ≤` `ATCG/ATCG` `< 19 ≤` `ATCG/ATCG` `< 33 ≤` `ATCG/ATCG` `< 38 ≤` `ATCG/ATCG`

Unaligned base: `atcg`    Masked matching base: `atcg`    Alignment gap: `‑`    Deleted base: `‑`

Reads not counted as support for junction

read\_name Not counted due to insufficient overlap past the breakpoint.

read\_name Not counted due to not crossing MOB target site duplication.
